# A Cu^2+^-Selective Probe Based on Phenanthro-Imidazole Derivative

**DOI:** 10.3390/s17010035

**Published:** 2016-12-24

**Authors:** Dandan Cheng, Xingliang Liu, Hongzhi Yang, Tian Zhang, Aixia Han, Ling Zang

**Affiliations:** 1 Chemical Engineering College, Qinghai University, Xining 810016, China; hanax@qhu.edu.cn (D.C.); liuxingliang@qhu.edu.cn (X.L.); yhz2011yj@163.com (H.Y.); 2 Qinghai Heavy Industry Vocational School, Xining 810101, China; 15897128508@163.com; 3 Department of Materials Science and Engineering, University of Utah, Salt Lake City, UT 84112, USA

**Keywords:** copper ion, fluorescent probe, phenanthro-imidazole

## Abstract

A novel fluorescent Probe **1**, based on phenanthro-imidazole has been developed as an efficient chemosensor for the trace detection of copper ions (Cu^2+^). Probe **1** demonstrated sensitive fluorescence quenching upon binding with Cu^2+^ through 1:1 stoichiometric chelation. The detection limit for Cu^2+^ ions was projected through linear quenching fitting to be as low as 2.77 × 10^−8^ M (or 1.77 ppb). The sensing response was highly selective towards Cu^2+^ with minimal influence from other common metal ions, facilitating the practical application of Probe **1** in trace detection.

## 1. Introduction

Copper (Cu^2+^), the third most abundant transition metal ion after Fe^2+^ and Zn^2+^ in the human body, plays a critical role in various fundamental physiological processes, such as those involving mitochondrial, cytosolic and vesicular oxygen-processing enzymes, which need copper as a redox cofactor [1,2]. Therefore, it is of great importance to develop simple, rapid and precise sensor methods to detect and monitor the concentration of Cu^2+^. Currently, there are many analytical methods for detecting Cu^2+^, such as atomic absorption spectroscopy (AAS) [3], inductive coupled plasma-mass spectroscopy (ICP-MS) [4], and fluorescence and surface plasmon resonance sensor methods [5,6,7]. Among these methods, fluorescence sensing remains one of the most promising approaches due to its high sensitivity, rapid response, and high selectivity through molecular binding design, as well as its simple solution assay processing [8,9,10,11,12]. Particularly, fluorescence sensors are suited for being embedded within tissues or cells for in situ imaging of Cu^2+^ ions and the associated physiological processes.

To date, numerous studies have been performed on the rational design of fluorescent chemosensors (probes) for the detection of ions and neutral analytes [13,14,15,16,17,18,19]. Many of these sensors have been proven effective for detecting Cu^2+^ ions [20,21,22,23,24,25,26,27,28], though in most of the cases the detection limit is not low enough to afford Cu^2+^ monitoring in blood and other biological systems. Moreover, the synthesis of fluorescence sensors often requires multiple step reactions, thus making the final product higher in cost, limiting the commercial use. To overcome these problems, we report herein on a novel fluorescent Probe **1**, which responds to the presence of Cu^2+^ with sensitive fluorescence quenching. Probe **1** is composed of a 1H-phenanthro [9, 10-d] imidazole moiety connected to a *N*,*N*-bis(pyridin-2-ylmethyl) benzeneamine unit, and can be synthesized in just one step. The selection of 1H-phenanthro [9, 10-d] imidazole dye is based on the consideration that it can function both as a fluorophore and an electron donor in an electron charge transfer (CT) system [12]. The *N*,*N*-bis(pyridin-2-ylmethyl) benzenamine moiety was chosen as the binding group for Cu^2+^ ions [29], and it can then become an efficient electron acceptor, resulting in fluorescence quenching through the CT process. Our study showed that the fluorescence quenching of Probe **1** was fast and highly selective towards Cu^2+^ over other common metal ions, implying great potential for using this probe for quick, trace-level detection of Cu^2+^ ions.

## 2. Experimental

### 2.1. Materials and Methods

All chemicals and reagents except for Probe **1** were used as purchased without further purification. For the synthesis of Probe **1** 300–400 mesh silica gel was used for column chromatography for the compound purification. ^1^H NMR and ^13^C NMR spectra were recorded on an Agilent DD2 NMR spectrometer (Agilent Technologies, Santa Clara, CA, USA) at 600 MHz, using DMSO-*d*_6_ as the solvent. Mass spectra were recorded on an Agilent Technologies 622 spectrometer (Agilent Technologies, Santa Clara, CA, USA). UV/vis spectra were acquired on a Shimadzu UV-2550 spectrophotometer (Shimadzu, Beijing, China). Fluorescence measurements were performed on an Agilent Cary Eclipse fluorescence spectrophotometer (Agilent Technologies, Santa Clara, CA, USA).

### 2.2. Synthesis

**Scheme 1:**
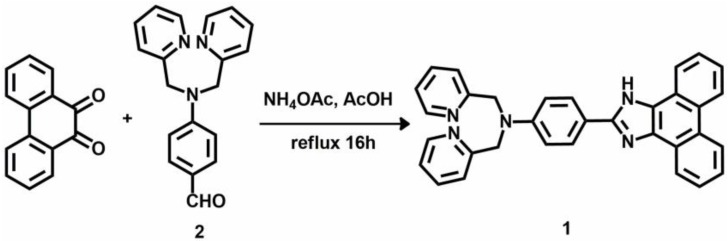
Synthetic route for fluorescent Probe **1**.

The synthetic route to Probe **1** is shown in Scheme 1. 1.5 g (4.93 mmol) Compound **2**, synthesized following the literature procedures [30], was mixed with phenanthrene-9, 10-dione (1.03 g, 4.93 mmol), and ammonium acetate (7.4 g, 98 mmol) in 63 mL acetic acid, and heated to reflux under nitrogen atmosphere for 16 h. The mixture was then cooled to room temperature and poured into H_2_O (100 mL), and the precipitate thus formed was filtered, washed with water and then dried under vacuum. The crude product obtained was purified via column chromatography (300–400 mesh silica gel), CH_2_Cl_2_/AcOEt, 4/1, v/v) to produce the desired product (1.263 g, 53% yield). ^1^H NMR (DMSO-*d*_6_, 600 MHz): δ = 8.77 (d, *J* = 8.4 Hz, 3H), 8.22 (d, *J* = 6 Hz, 2H), 7.91 (d, *J* = 7.2 Hz, 1H), 7.64 (t, *J* = 7.2 Hz, *J* = 7.2 Hz, 4H), 7.59–7.55 (m, 4H), 7.52 (t, *J* = 7.2 Hz, *J* = 7.8 Hz, 1H), 7.31–7.25 (m, 4H), 6.99 (d, *J* = 7.2 Hz, 1H), 4.08 (s, 1H), 2.49 (d, *J* = 1.8 Hz, *J* = 1.8 Hz, 4H). (Figure S1, Supplementary Information). ^13^C NMR (DMSO-*d*_6_, 150 MHz) δ 141.34, 130.39, 130.02, 129.50, 128.79, 128.44, 127.40, 127.28, 127.22, 126.99, 126.93, 125.01, 124.95, 123.73, 121.52, 48.49 (Figure S2, Supplementary Information). MALDI-TOF MS: *m/z* calculated for C_33_H_25_N_5_: 491.2110; found: 491.2141 (Figure S3, Supplementary Information).

### 2.3. Spectral Measurements

Distilled water was used for preparing solutions throughout the experiments. Solutions of all the metal ions used (Cu^2+^, Hg^2+^, Ca^2+^, Ba^2+^, Cd^2+^, Zn^2+^, Pb^2+^, Mg^2+^, Co^2+^, Fe^2+^ and Mn^2+^) were prepared from their nitrate salts. A stock solution (0.5 mM) of Probe **1** in ethanol was prepared, which was then diluted to 10 μM with ethanol. In the spectral titration experiments, 2 mL of Probe **1** solution (10 μM) was placed in a 1 cm quartz cuvette and Cu^2+^ solution was added gradually by micro-pipette; UV-vis and/or fluorescence spectra were measured before and after the addition of Cu^2+^. Since the volumes of Cu^2+^ solution added were minimal (in μL), the slight change in concentration of Probe **1** can be ignored. For fluorescence measurement, the excitation wavelength was set at 270 nm, and the slit widths for excitation and emission were 5 nm/5 nm.

## 3. Results and Discussion 

### 3.1. UV-Vis Spectral Response of the Binding between Probe **1** and Cu^2+^

**Scheme 2:**
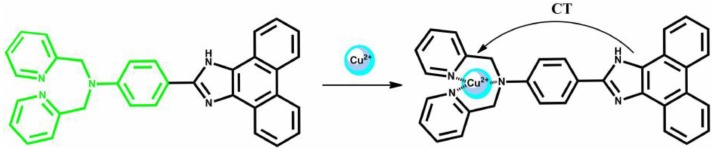
Chelation with Cu^2+^ quenches the fluorescence of Probe **1** via intramolecular CT.

**Figure 1:**
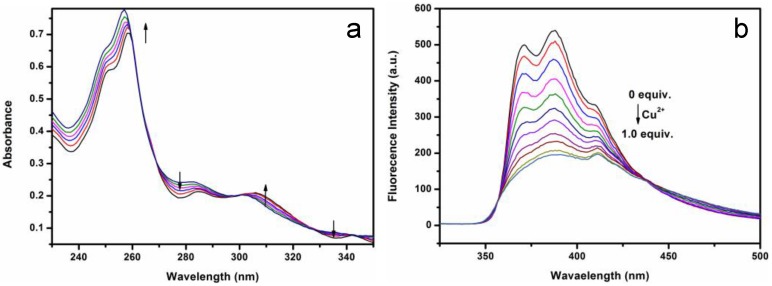
UV/vis absorption (**a**) and fluorescence (**b**) spectra of Probe **1** in ethanol (10 μM) upon addition of varying concentrations of Cu^2+^ ions (0–1 equiv).

Probe **1** binds effectively with Cu^2+^ ions through the chelation with *N*,*N*-bis(pyridin-2-ylmethyl) benzenamine (Scheme 2). The same chelation was previously reported in a crystalline study of the complex of Cu^2+^ [29]. The strong complexation affects the original conjugation between the lone pair of electrons on the aniline amine and the π-orbital of 1H-phenanthro [9, 10-d] imidazole. This can be seen from the significant change in the absorption spectrum of Probe **1** as shown in Figure 1a. Upon the addition of a Cu^2+^ ion, a significant absorption increase was observed for the wavelength region below 260 nm and in the region between 300 and 325 nm, whereas the absorption in the range of 270–300 nm and 325–345 nm was decreased. Clear isosbestic points can be identified at 269, 300, 328 and 343 nm between the increasing and decreasing bands, indicating the stoichiometric chelation equilibrium shown in Scheme 2. 

Along with the absorption change, the fluorescence spectra recorded accordingly also demonstrated a significant change as shown in Figure 1b. The fluorescence quantum yield of Probe **1** in the absence of Cu^2+^ was determined to be 6.7%, which represents a medium-strength fluorophore suited for being used as a sensor. Upon the addition of a 1:1 molar ratio of Cu^2+^ ions, the fluorescence intensity was quenched by 74%. Interestingly, the fluorescence quenching was dominated by the emission in the shorter wavelength region, while the emission at longer wavelengths (above 437 nm) was actually increased slightly, implying the formation of a charge transfer (CT) transition between the 1H-phenanthro [9, 10-d] imidazole moiety and the Cu^2+^ complex. The fluorescence quenching observed was likely due to the photoinduced electron transfer from the lowest unoccupied molecular orbital (LUMO) of 1H-phenanthro [9, 10-d] imidazole to the Cu^2+^ ion.

### 3.2. Stoichiometric Ratio of Probe **1**-Cu^2+^ Complex

**Figure 2:**
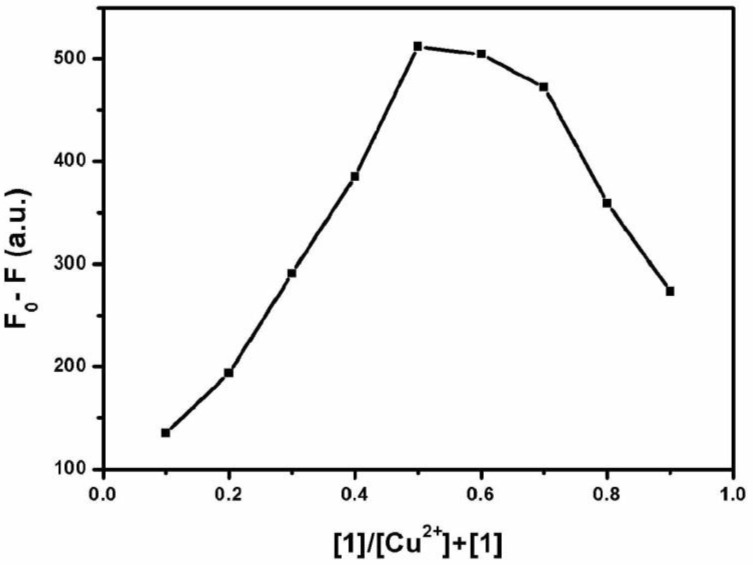
Job plot of the binding between Probe **1** and Cu^2+^ in ethanol, with the total concentration of the two kept constant at 10 μM. F_0_ and F are fluorescence intensities measured at 387 nm in the absence and presence of Cu^2+^, respectively.

The sensitive fluorescence quenching of Probe **1** by the Cu^2+^ ions provided a way to determine the chelation stoichiometry between the two species simply through a Job plot approach, as shown in Figure 2 [31]. A Job plot is commonly used to determine the stoichiometry of a binding event between two species in a solution. In this method, the total molar concentrations of the two binding species (here Probe **1** and Cu^2+^ ions) are held constant, while their molar fractions are varied. An observable variable (here the fluorescence quenching) that is proportional to the complex formation can be plotted against the molar fractions of the binding species. The maximum of the plot corresponds to the stoichiometry of the complex formed by the two binding species. In this study, by fixing the total concentration of Probe **1** and the Cu^2+^ ions at 10 μM, the molar ratio of the two species was changed from 1:9 to 9:1, and the fluorescence intensity of the mixture was measured at 387 nm under the same conditions. The molar ratio that gives the maximal fluorescence quenching should correspond to the stoichiometry between Probe **1** and Cu^2+^ ions, ca. 1:1 as indicated in Figure 2. The 1:1 ratio is consistent with the previous reports on the same chelation of *N*,*N*-bis(pyridin-2-ylmethyl) benzenamine with Cu^2+^ ions [29]. 

### 3.3. Fluorescence Quenching Selectivity

**Figure 3:**
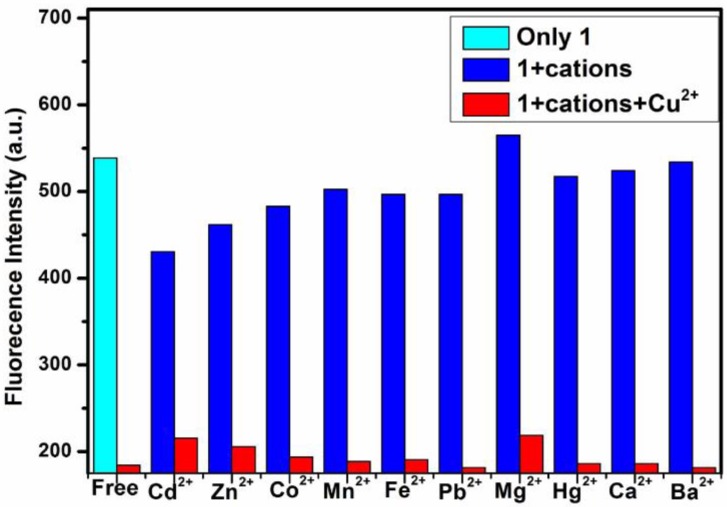
Fluorescence intensity measured at 387 nm for Probe **1** in ethanol (10 μM) in the absence of metal ions (**Green**), and in the presence of 10 μM various metal ions (**Blue**); 10 μM Cu^2+^ was added to each of the 11 solutions and the fluorescence intensity was measured again for comparison (**Red**).

To examine the fluorescence quenching selectivity of Probe **1** towards Cu^2+^, comparative experiments were conducted for the same quenching but in the presence of 10 other common metal ions, as shown in Figure 3 and Figures S5 and S6. Compared to the efficient quenching by Cu^2+^ (far left bar in the figure), all other metal ions gave a much lower degree of quenching under the same experimental conditions. Adding the same concentration of Cu^2+^ to each of the 10 solutions containing the different metal ions resulted in dramatic fluorescence quenching at the same level as that observed for the solution containing only Cu^2+^ as the quencher. These results indicate good selectivity for Probe **1** towards Cu^2+^ when used as a fluorescence sensor. The high selectivity is due to the strong chelation interaction between Probe **1** and Cu^2+^ as shown in Scheme 2, as well as the photoinduced electron transfer thus enabled between the two species. Although Probe **1** also binds to other metal ions such as Co^2+^, Zn^2+^, Cd^2+^, these ions do not possess a strong electron-accepting capability as Cu^2+^ does, and thus can hardly induce effective photoinduced electron transfer. The stronger electron acceptability of Cu^2+^ can be seen from its higher standard reduction potential, +0.34 V, much higher than those of Co^2+^, Zn^2+^, Cd^2+^, −0.29, −0.70, −0.40 V, respectively.

### 3.4. Detection Limit

**Figure 4:**
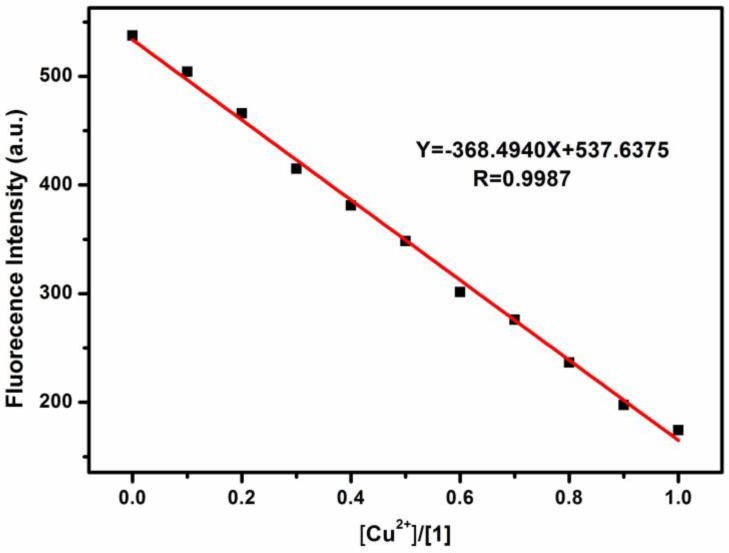
Fluorescence intensity measured at 387 nm for Probe 1 in ethanol (10 μM) as a function of the concentration of Cu^2+^ (0–1 equiv.). Inset shows the linear fitting result of the data points.

Figure 4 shows the fluorescence intensity of Probe **1** (10 μM in ethanol) as a function of the concentration of Cu^2+^ (plotted here as the ratio of [Cu^2+^]/[1]). All the data points can be fitted well into a linear relationship, giving the equation as marked in the plot (with a slope of 368.49). Following the common practice in analytical chemistry, the detection limit can be calculated by defining the lowest detectable signal as three times the standard deviation of the intensity measurement. In this study, the standard deviation of the intensity measurement was 0.34, and three times that gives 1.02. This value represents the minimal detectable change in the fluorescence intensity, which corresponds to the lowest detectable value of [Cu^2+^]/[1] (calculated as 1.02/slope = 2.77 × 10^−3^). Since the concentration of Probe **1** was kept at 10 μM, the detection limit of Cu^2+^ was obtained as 2.77 × 10^−8^ M (or 1.77 ppb). Such a low detection limit is significantly improved, by one to three orders of magnitude, in comparison to the previously reported chemosensors (Figure S7). A low detection limit will be suitable for the trace detection of Cu^2+^ in blood [32].

In addition to the high sensitivity and selectivity, a fast sensing response was another feature of Probe **1** regarding the detection of Cu^2+^. Upon the addition of an equivalent amount Cu^2+^ ions, the fluorescence intensity of Probe **1** (10 μM) in ethanol was quenched rapidly (Figure S4), with a response time estimated to be ca. 10 s (inset of Figure S4). This fast sensing response makes Probe **1** highly suited for real-time monitoring, or portable detection [33], which is not feasible for the traditional analytical methods and many other chemosensors reported before. Moreover, Probe **1** was also proven to have high photostability as shown in Figure S8, wherein the fluorescence of Probe **1** was measured multiple times over 2 h, but no significant decrease in the fluorescence intensity was observed. 

## 4. Conclusions

In conclusion, we have developed an efficient molecular fluorescence sensor, Probe **1**, based on phenanthro-imidazole for quick trace-level detection of Cu^2+^ ions in aqueous solutions. Probe **1** demonstrated sensitive fluorescence quenching upon binding with Cu^2+^ ions through 1:1 stoichiometric chelation. The detection limit was projected through linear quenching fitting to be as low as 2.77 × 10^−8^ M (or 1.77 ppb), which is improved by one to three orders of magnitude in comparison to the previously reported chemosensors. The fluorescence sensing response was highly selective towards Cu^2+^ ions without significant interference from other common metal ions under the same conditions. The sensing response towards Cu^2+^ ions was also found quickly, on the time scale of seconds. Moreover, high photostability was also proven for Probe **1** by repeatedly measuring the florescence over 2 h. The combination of all these features makes Probe **1** an ideal sensor for the portable, real-time detection of copper ions. 

## Supplementary Materials

The following are available online at http://www.mdpi.com/1424-8220/17/1/35/s1, Figure S1: ^1^H NMR (600 MHz) spectrum of compound 1 in d-DMSO; Figure S2: ^13^C NMR (150 MHz) spectrum of compound 1 in d-DMSO; Figure S3: MALDI/TOF MS spectrum of compound 1; Figure S4: Fluorescence intensity measured at 387 nm for probe 1 in ethanol (10 μM) as a function of time upon addition of Cu^2+^ (10 μM). Exponential fitting of the fluorescence intensity decrease gives a response time of ca. 10 s; Figure S5: Fluorescence spectra of probe 1 in ethanol (10 μM) in the absence and presence of various metal ions (10 μM); Figure S6: Fluorescence spectra of probe 1 in ethanol (10 μM) in the absence and presence of various metal ions (10 μM) plus 10 μM of Cu^2+^; Figure S7: Comparison of the detection limit of Probe 1 with the literature reported detection limits of other sensors. Reference # marked in the horizontal axis are the same as cited in the main context; Figure S8: Fluorescence intensity measured at the main peak of probe 1 in ethanol (10 μM) for nine consecutive times over 2 h.
