# Supplementary material for: A Cu2+-Selective Probe Based on Phenanthro-Imidazole Derivative
Source: Sensors (Basel). 2016 Dec 24;17(1):35. doi: 10.3390/s17010035 (PMC5298608; doi:10.3390/s17010035)
Supplement: Supplementary file 1 [file sensors-17-00035-s001.pdf]

Supplementary Materials: A Cu^{2+}-Selective Probe Based on Phenanthro-Imidazole Derivative

Dandan Cheng, Xingliang Liu, Hongzhi Yang, Tian Zhang, Aixia Han and Ling Zang

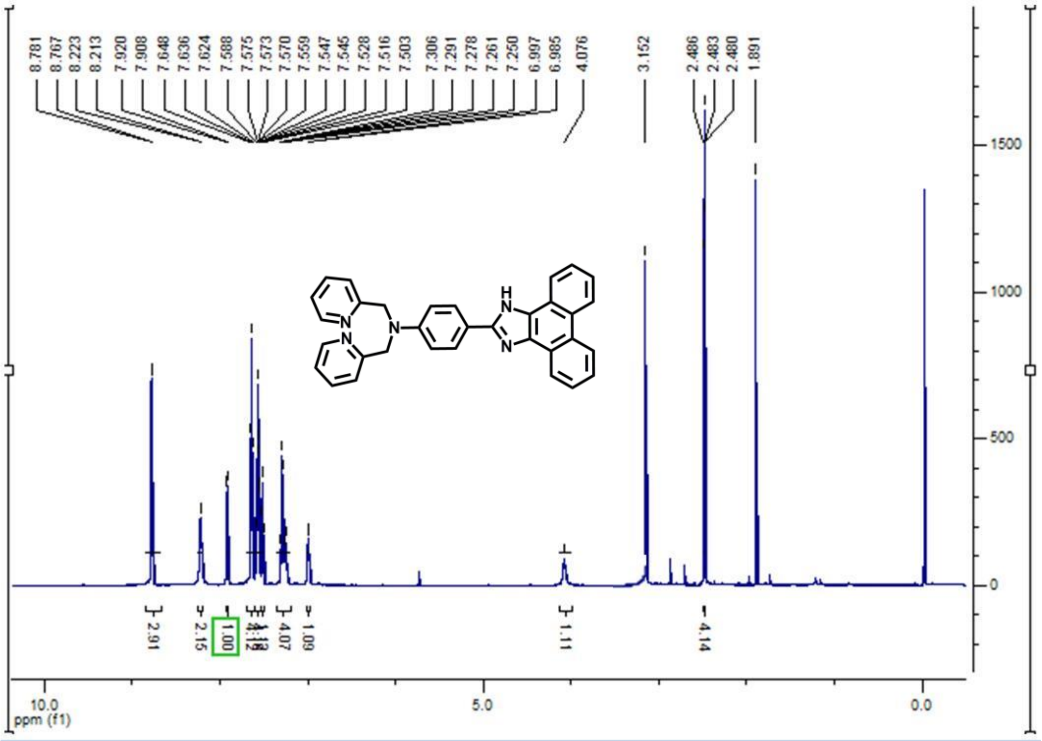

Figure S1. ^{1}H NMR (600 MHz) spectrum of compound **1** in d-DMSO.

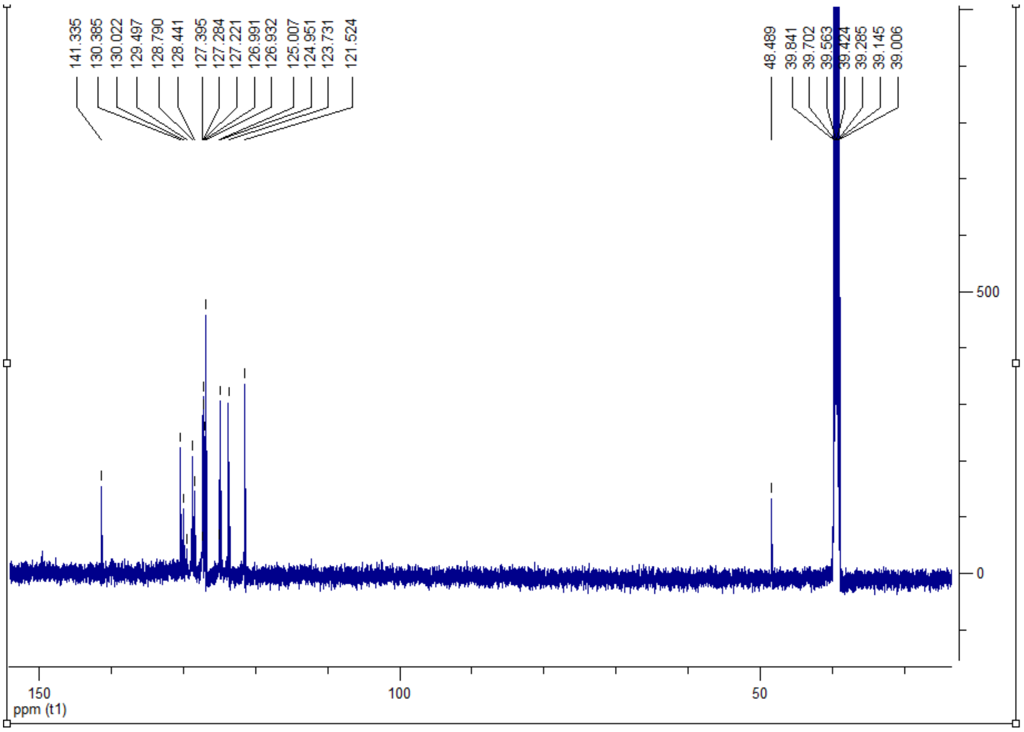

Figure S2. ^{13}C NMR (150 MHz) spectrum of compound **1** in d-DMSO.

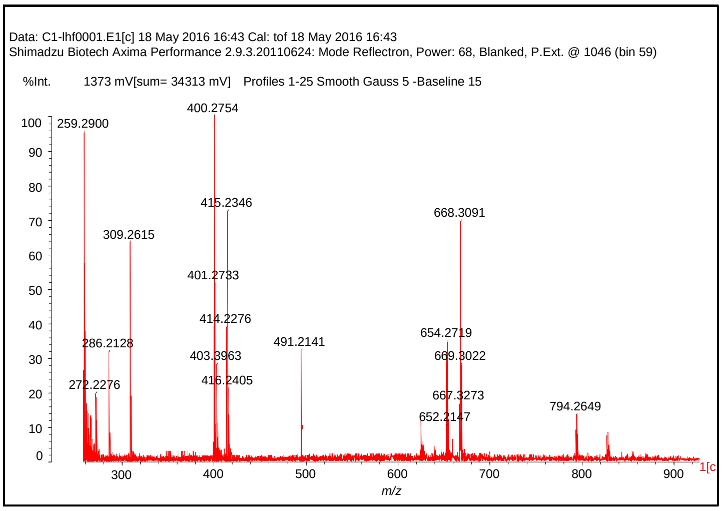

Figure S3. MALDI/TOF MS spectrum of compound **1**.

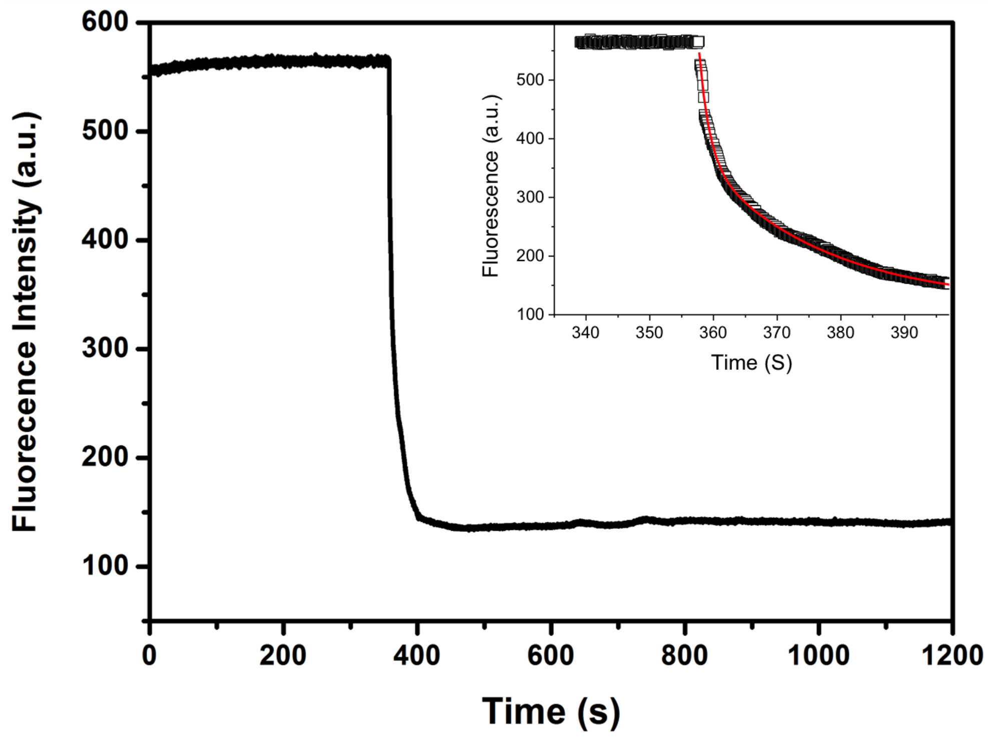

Figure S4. Fluorescence intensity measured at 387 nm for probe **1** in ethanol (10 μM) as a function of time upon addition of Cu^{2+} (10 μM). Exponential fitting of the fluorescence intensity decrease gives a response time of ca. 10 s.

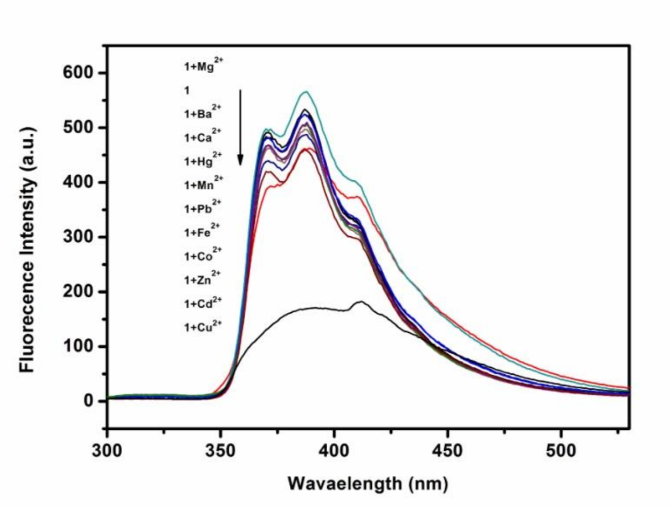

Figure S5. Fluorescence spectra of probe **1** in ethanol (10 μM) in the absence and presence of various metal ions (10 μM).

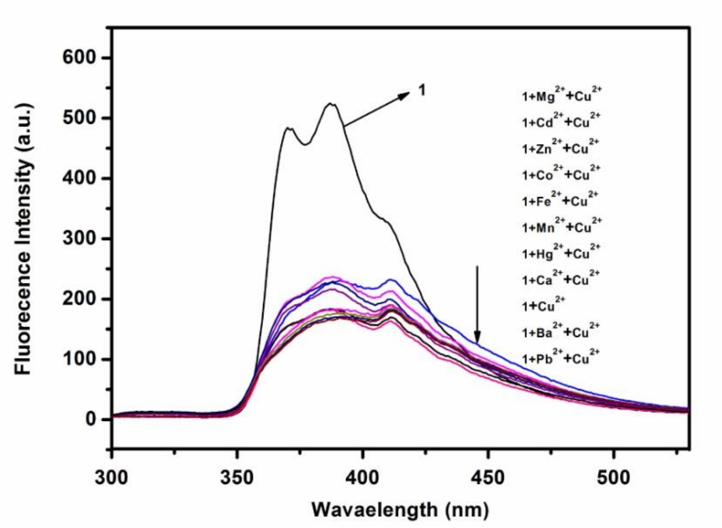

Figure S6. Fluorescence spectra of probe **1** in ethanol (10 μM) in the absence and presence of various metal ions (10 μM) plus 10 μM of Cu^{2+}.

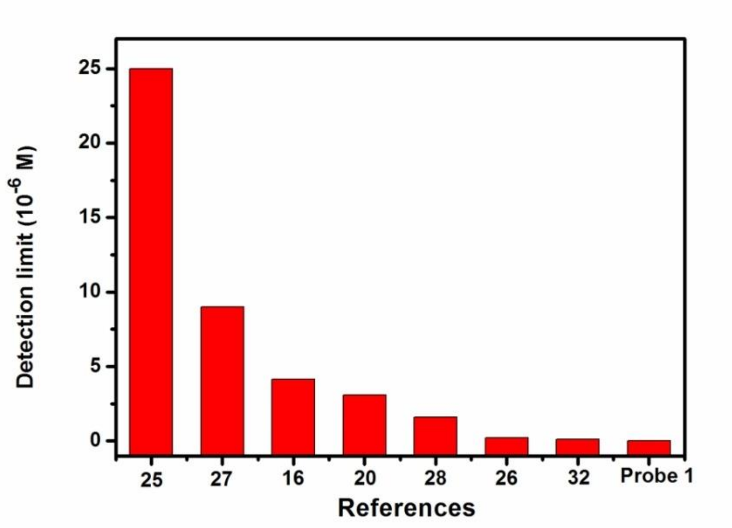

Figure S7. Comparison of the detection limit of Probe 1 with the literature reported detection limits of other sensors. Reference # marked in the horizontal axis are the same as cited in the main context.

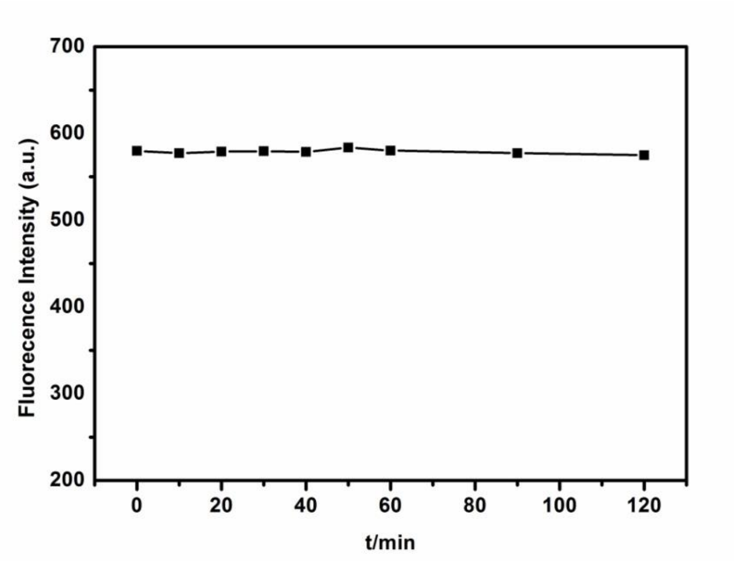

Figure S8. Fluorescence intensity measured at the main peak of probe **1** in ethanol (10 μM) for nine consecutive times over 2 h.
